# Expression of NEDD9 and connexin-43 in neoplastic and stromal cells of gastric adenocarcinoma

**DOI:** 10.17305/bjbms.2020.5379

**Published:** 2021-10

**Authors:** Ivan Lerotić, Petra Vuković, Davor Hrabar, Zvonimir Misir, Ivan Kruljac, Jelena Forgač, Petra Ćaćić, Tajana Pavić, Monika Ulamec

**Affiliations:** 1 Department of Gastroenterology and Hepatology, University Hospital Center “Sestre milosrdnice”, Zagreb, Croatia; 2 Department of Medical Oncology, University Hospital for Tumors, University Hospital Center Sestre Milosrdnice, Zagreb, Croatia; 3 School of Medicine, University of Zagreb, Zagreb, Croatia; 4 Department of Surgery, University Hospital Center “Sestre milosrdnice”, Zagreb, Croatia; 5 Department of Endocrinology, Diabetology and Metabolic diseases “Mladen Sekso”, University Hospital Center “Sestre milosrdnice”, Zagreb, Croatia; 6 Department of Pathology and Cytology “Ljudevit Jurak”, University Hospital Center “Sestre milosrdnice”, Zagreb, Croatia; 7 School of Dental Medicine, University of Zagreb, Zagreb, Croatia; 8 Scientific Group for Research on Epigenetic Biomarkers, University of Zagreb, Zagreb, Croatia

**Keywords:** Gastric cancer, NEDD9, conexin-43, microenvironment, metastases

## Abstract

Gastric cancer is related to high mortality rates and advanced disease stage at the time of diagnosis. Its carcinogenesis is extensively studied and is associated with genetic and epigenetic changes, changed the interaction between tumor and adjacent stromal cells, and changes in the microenvironment molecule status. Neural precursor cell-expressed developmentally down-regulated 9 (NEDD9) affects different signaling proteins and pathways, apoptosis, adhesion, cell migration, and invasiveness. Connexin-43 (Cx43) also assists in intercellular communications and has several channel-independent functions. Aberrant expression of those two gap junction proteins plays an essential role in metastatic processes. Our scope was to detect the expression of Cx43 and NEDD9 in epithelial and stromal gastric cancer compartments and its relation to tumor progression and lymph node metastases. Cancer tissue from 53 cases of node-negative and 55 cases of node-positive primary gastric carcinoma patients was analyzed for Cx43 and NEDD9 expression by immunohistochemical assay, and the results were correlated with the remaining clinical and pathological findings and survival. In our cohort of patients with lymph node metastases, we detected higher expression of epithelial Cx43 in the primary tumor and stromal Cx43 expression correlated with both epithelial NEDD9 (rho = 0.453) and stromal NEDD9 (rho = 0.484). Higher epithelial Cx43 and NEDD9 expression were associated with higher mortality (HR 1.54, 95% CI 1.01-2.37, p = 0.048). Epithelial Cx43 expression, both epithelial and stromal NEDD9 expression, T and N status were all independently associated with shorter survival. In summary, our findings suggest that increased expression of both epithelial and stromal NEDD9 and epithelial Cx43 could potentially be used as prognostic gastric cancer biomarkers.

## INTRODUCTION

Gastric cancer is the fifth most common malignancy and the third most common cause of cancer death in the world [1,2]. Eastern Europe is one of the regions with the highest incidence of gastric cancer alongside with East Asia and South America. High mortality is associated with the fact that it is frequently diagnosed at the advanced stage of the disease [1-3].

Invasion and metastasis are one of the hallmarks of cancer, which are responsible for the greatest number of cancer-related deaths [4,5]. This complex process is a sequence of several steps, invasion-metastasis cascade, intravasation, transit of cancer cells in circulation, and extravasation and colonization. Those are all processes in which cancer cells are interacting with recruited and activated normal stromal and inflammatory cells to create suitable tumor microenvironment, which plays an important role in the tumor behavior and prognosis [5,6]. Gap junction proteins are specialized membrane structures that allow the formation of channels between cells enabling communication and transfer of ions and small molecules <1 kDa [7]. Gap junction proteins have an important role in intercellular communication, adhesion, and cell proliferation and differentiation. Their aberrant expression has role in carcinogenesis, invasion, and metastasis [6-8].

Connexin-43 (Cx43) is the ubiquitous and intensively studied connexin protein. In addition to its function as s gap-junction-channel-forming protein, Cx43 also assists in other types of intercellular communications, including communication between non-opposed cells through tunneling nanotubes and extracellular vesicles. Cx43 also has several channel-independent functions such as modulation of cell adhesion, differentiation, proliferation, and gene transcription [8,9]. Neural precursor cell-expressed developmentally down-regulated 9 (NEDD9) (also known as HEF1/Cas-L) is a member of the Cas protein family of signal transporters [10]. In addition to its enzymatic protein activity, NEDD9 contains several interaction domains through which it interacts and affects other signaling proteins and signaling pathways, apoptosis, adhesion, cell migration, and invasiveness [11-14]. It is suggested that aberrant expression of these two gap junction proteins, Cx43 and NEDD9, plays an important role in metastasis process in different tumors. Role of and connection between those molecules in gastric cancer are not entirely understood [15].

The aim of this study was to determine the expression of Cx43 and NEDD9 in node-positive and node-negative gastric cancer at gastrectomy tissue, to establish its association with relevant clinical and pathological parameters, with emphasis on prognosis.

## MATERIALS AND METHODS

Materials from 108 patients with gastric cancer without prior therapy in the period from January 1, 2005 till November 31, 2015, were retrieved from Ljudevit Jurak Pathology Department Tumor Registry, Zagreb, Croatia. Materials consisted of total gastrectomy with D2 lymphadenectomy specimens; there were 53 patients with N0M0 disease and 55 patients with N1M0 disease.

Tissue for light microscopy was fixed in 4% formaldehyde, embedded in paraffin using routine procedures, from which 5 mm thin sections were cut and stained with hematoxylin and eosin (HE). All cases were diagnosed by pathologists and meet the WHO criteria of gastric adenocarcinoma, NOS. Representative block with tumor tissue was selected for each patient; tissue microarray was performed and analyzed on HE slides. Tumors were divided into two groups according to lymph node status (negative/positive).

The following primary polyclonal rabbit antibodies were employed: NEDD9 (1:400, Abcam, Cambridge, UK) and Cx43 (1:300, Santa Cruz Biotechnology, Santa Cruz, California). DAKO EnVisionTM+System, HRP (DAB) was used to visualize positive reactions according to the manufacturer’s instructions. The slides were counterstained with hematoxylin. Appropriate negative and positive controls (colon mucosa for NEDD9 and C43) were employed. Immunohistochemical staining was evaluated for the whole section with minimum of 1000 tumor cells (TCs).

Morphometric analysis for protein expression of investigated genes was performed by two pathologists. All disagreements were resolved by a joint committee. Expression of proteins was analyzed in two compartments, i.e. epithelial and stromal cells. Staining signal (brown in color) was noted as cytoplasmic or membranous. Staining percentage was scored from 0 to 3; 0 (negative TCs); 1 (up to 10% positive TC); 2 (from 10% to 50% positive TC); and 3 (more than 50% positive TC). Intensity of staining was assessed as 0-negative; 1-low, 2-medium, and 3-high. Semi-quantification of protein expression was expressed by immunoreactivity score (IRS) which was calculated by multiplying staining percentage (0-3) and intensity of staining (0-3) creating a range of 0-9. IRS score 0 was considered negative; up to 6 was considered low and 6-9 high.

Clinical and histopathological data (age, gender, tumor size, tumor type, TNM, vascular invasion, perineural invasion, and median survival) were retrieved from the University Hospital Center Sestre milosrdnice patient data archive and from the Croatian Institute of Public Health, respecting all ethical permissions for this study.

### Ethical statement

Ethical agreements were obtained from the ethics committee of the Medical Faculty University of Zagreb (641-01/21-02/01), as well as the University Hospital Center Sestre milosrdnice (EP-7407/15-19).

### Statistical analysis

Patient characteristics were assessed using descriptive statistics. Continuous variables were expressed as median with interquartile range and compared with Mann–Whitney and Kruskal–Wallis test when appropriate. Categorical variables were analyzed using the Chi-square test with Yates correction. Spearman correlation coefficients were used for univariate analysis of coherence between immunohistochemistry parameters. Kaplan–Maier curves and Log-Rank tests were used to analyze the difference in patient outcomes. Multivariate Cox regression analysis was used to determine which parameters were independently associated with survival. Backward stepwise conditional approach was used in multivariate analysis.

Receiver operating characteristic analysis was carried out to analyze the predictive capacity of each variable in predicting 2-year survival.

Finally, a *post-hoc* calculation of achieved power was performed with G-power software version 3.1.9.7. We used an exact test family with a method of inequality, two independent groups (Fisher’s exact test). Input parameters for the aforementioned calculation were retrieved from the difference in 2-year survival between patients with positive and negative epithelial NEDD9 expression.

Two-sided *p* < 0.05 were considered significant. The statistical analysis was done using IBM SPSS Version 20.0.

## RESULTS

### Univariate analyses

In our cohort overall median age was 68 years (59-75) and median survival was 31.0 (13.318-48.682) months, respectively.

Patients with positive lymph nodes were younger, had larger primary tumors with higher rates of perineural and vascular invasion. Similar observations were made after analysis of the aforementioned parameters based on stage of the disease, except for patient age and tumor type (Table 1). In respect to the expression of Cx43 and NEDD9, patients with lymph node metastases showed higher expression of epithelial Cx43 in the primary tumor (Table 1).

**TABLE 1:**
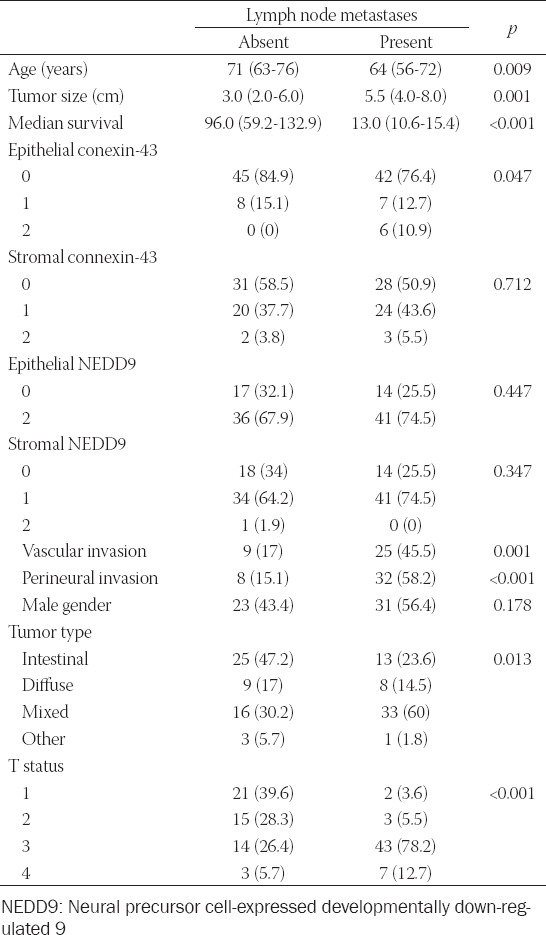
Univariate analysis of the study population based on lymph node metastases

**FIGURE 1:**
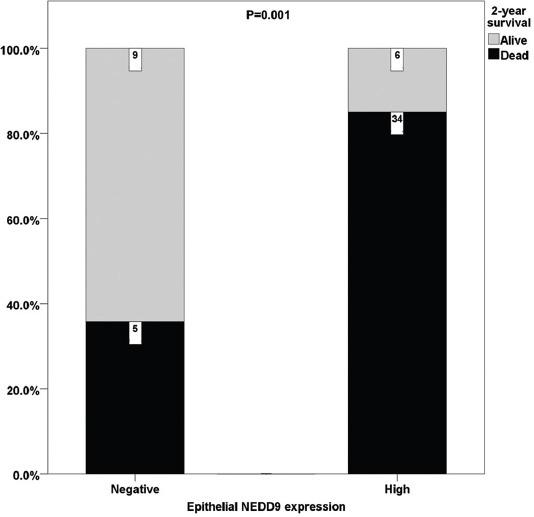
Two-year survival rate in patients with lymph node metastases correlated to epithelial neural precursor cell-expressed developmentally down-regulated 9 expression.

Stromal Cx43 expression correlated with both epithelial NEDD9 (rho = 0.453) and stromal NEDD9 (rho = 0.484). Although we have found strong correlation between the stromal and epithelial NEDD9 expression (rho = 0.967), similar association between stromal and epithelial Cx43 expression has not been found (rho = 0.100, *p* = 0.305) (Figure 1).

Higher epithelial Cx43 expression was associated with higher mortality (HR 1.54, 95% CI 1.01-2.37, *p* = 0.048), as was epithelial NEDD9 expression (HR 1.55, 95% CI 1.16-2.07, *p* = 0.03). Stromal NEDD9 expression has not been further analyzed due to high correlation with epithelial NEDD9 expression. We found no association between the stromal Cx43 expression and survival.

### Multivariate models

In a multivariate analysis consisting of age, gender, tumor type, tumor size, vascular and perineural invasion (Model 1), tumor size and perineural invasion were found to be independently associated with survival.

When we removed tumor size and included T and N status into the model (Model 2), T and N status were only two parameters associated with survival. When epithelial and stromal expression of Cx43 and NEDD9 were the sole parameters of the multivariate model (Model 3), epithelial and stromal expression of NEDD9 were associated with shorter survival. When we included epithelial and stromal expression of Cx43 and NEDD9 into Model 1 (Model 4), we found that both epithelial and stromal expression of NEDD9 remained to be associated with shorter survival. Finally, epithelial and stromal expression of Cx43 and NEDD9, T status and N status were included in a separate model (Model 5). Epithelial Cx43 expression, both epithelial and stromal NEDD9 expression, T and N status were all independently associated with shorter survival. All models have been presented in Table 2.

**TABLE 2:**
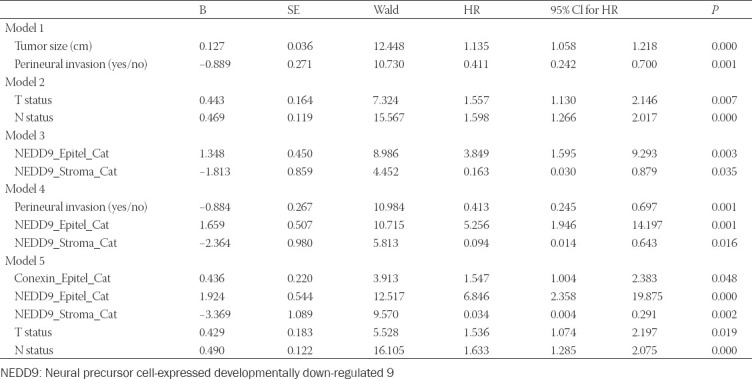
Multivariate analysis models

### Predicting 2-year survival

Tumor size, age, T status, and N status could all predict 2-year survival, but N status had the best diagnostic accuracy (79.0%). Epithelial NEDD9 could predict 2-year survival with 65.7% accuracy, while epithelial Cx43 expression had no predictive capacity (Table 3).

**TABLE 3:**
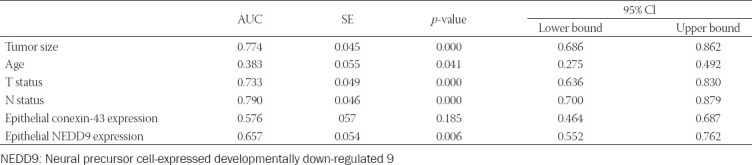
Accuracy of each variable in predicting 2-year survival

**FIGURE 2:**
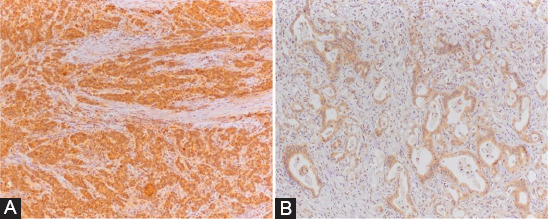
Immunohistochemical staining; (A) strong epithelial neural precursor cell-expressed developmentally down-regulated 9 (NEDD9) expression without stromal expression (NEDD9 × 200), (B) strong epithelial connexin-43 (Cx43) expression with low stromal expression (Cx43 × 200).

When we analyzed differences in 2-year survival, high expression of both epithelial Cx43 and NEDD9 was more prevalent in patients who died. In a subgroup analysis of patients with and without lymph node metastases, there were no significant differences in Cx43 and NEDD9 expression in patients without lymph node metastases, but patients with lymph node metastases showed higher expression of epithelial NEDD9 (Table 4 and Figure 2).

**TABLE 4:**
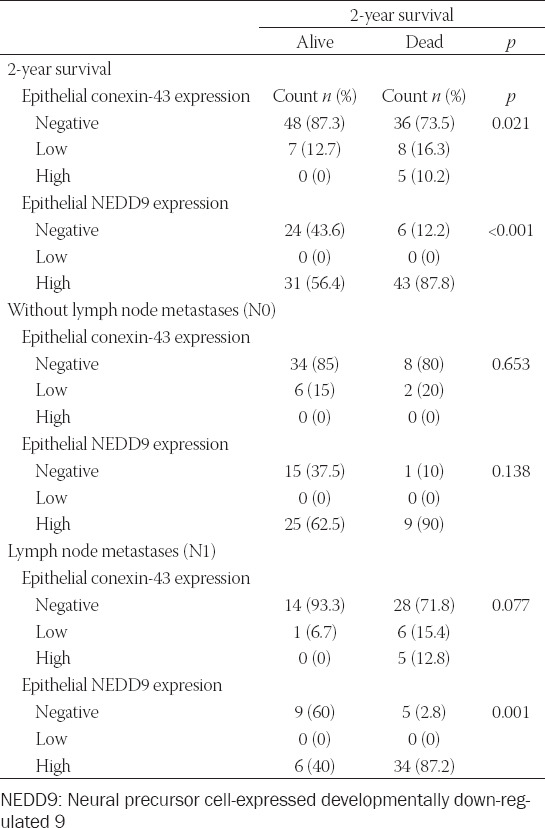
Expression of immunohistochemical parameters in relation to 2-year survival and lymph node metastases

Finally, a *post-hoc* calculation of achieved power was performed. Two-year mortality in patients with negative NEDD9 expression was 35.7% (5/14) and 85.0% (34/40) in patients with positive NEDD9 expression. Consequently, a power of our sample size was 93.8% with an actual a 2.4%.

## DISCUSSION

Interaction between epithelial TCs, as well as in between epithelial and stromal cells are key to the tumor proliferation, dedifferentiation, local, and metastatic spread. Those interactions are connected to the change of cell microenvironment [15]. NEDD9 is important component of integrin-dependent signaling cascade that activates FAK and Src kinases to promote cell migration and contribute to normal mitotic progression, mediates proliferative, and survival signaling [8,10]. NEDD-9 is acting as pro-metastatic stimulus in a several cancer types changing tumor microenvironment by influencing cell attachment, migration, invasion, cell cycle, apoptosis, and oncogenic signal transduction [10]. The increased expression of NEDD9 has been associated with the aggressiveness of lung, breast, prostate cancer and melanoma, as well as CNS, and gynecological cancers [14,16-19]. Similar as in our study, expression of NEDD9 was found significantly increased in the tissue of pancreatic ductal adenocarcinoma compared to adjacent pancreatic tissue [18], but the same was not established in colorectal cancer [19].

Several studies in the Asian population have linked increased expression of NEDD9 with more aggressive clinical course of gastric cancer [20-24]. It was correlated to the tumor size, vascular and stromal invasion, as well as lymph node, and distant organ metastases. Multivariate analysis found expression of NEDD9 and FAK as independent prognostic indicators for gastric cancer [21-23].

In our study, 78% of patients had high epithelial NEDD9 expression while stromal NEDD9 expression was absent or low. Our results are in concordance with previous results investigating association of increased expression of NEDD9 in gastric cancer with lymph node positivity. We also found that increased expression of both, epithelial and stromal NEDD9 is related to poor prognosis.

Interestingly, preclinical study on mice from Zhang et al. showed that inhibition of NEDD9 mRNA and protein expression by small interfering RNA (siRNA) could inhibit proliferation, migration, and invasion of BGC823 cells [24]. This, along with the fact that NEDD9 is highly expressed in gastric cancer tissue compared to healthy tissue (92.5 vs. 7.5%) [23] indicates that NEDD9 could potentially serve as target for future therapy strategies in gastric cancer.

In our study, we were also interested in expression of Cx43 on both, epithelial and stromal gastric cancer cells and its possible link to NEDD9. Connexins expression is often changed within various tumor types and Cx43 is one of the most commonly related to changes in microenvironment. In some cancer types increased Cx43 expression is correlated to favorable outcome, such as in breast, colorectal, and prostate cancer. It is connected with loss of gap junction intercellular communication in colorectal cancer [25], which results in downregulation, or aberrantly localized Cx43 and may predict clinical outcome. Similar findings are seen in the prostate and breast cancer, as well as in advanced non-small cell lung cancer [25-29]. Tang et al. showed that expression of Cx43 was significantly reduced in primary gastric cancer tumors, compared to adjacent normal tissues, but significantly increased in metastatic lymph nodes, compared to primary tumor [30]. Our results showed somewhat different expression. In general, Cx43 expression was absent or low in the epithelial component of cancer but subset of lymph node metastatic tumors showed increased epithelial Cx43 expression in primary tumor which was related to higher mortality. In the same work, Tang et al. found that reduced expression of Cx43 was correlated with poor differentiation and advanced TNM stage but they did not observe any significant difference in survival based on the expression of Cx43. In a different study, the same group speculated that connexins may play important role in the extravasation of cancer cells into lymphoid tissue forming gap junctions between TCs and endothelial cells in lymph node vessels and that their finding reflects higher degree of differentiation in metastatic cells compared with cells in the primary tumor site [30,31]. Recent study of Li et al. showed that expression of Cx43 was lower, both on protein and mRNA level, in gastric cancer tissue compared to healthy gastric tissue (*p* < 0.05) and that expression of Cx43 was lower in gastric cancers with metastasis than those without lymph node metastasis [32]. Furthermore, Kim et al. showed that low expression of Cx43 was associated with worse cancer-specific survival in patients with gastric cancer after gastrectomy [33].

Other studies show completely different results in Cx43 expression and increased expression is associated with poor prognosis in bladder, esophageal squamous cell, and oral squamous cell cancer and melanoma [34-37].

Our study has several limitations, the major one being the retrospective nature of the study with relatively small sample size from single institution. Evaluation of NEDD9 and Cx43 was performed on protein level by immunohistochemistry and data on differences in subsequent oncological treatments were not included. Indeed, the role of connexins in cancer and cancer progression seems to be controversial. Aberrant expression of connexins, both up- and downregulation can contribute to cancer development and progression and Cx43 may act as both; oncogene and tumor suppressor [38].

## CONCLUSION

Epithelial Cx43 expression, both epithelial and stromal NEDD9 expression, T and N status were all independently associated with survival in gastric cancer patients. Epithelial NEDD9 could predict 2-year survival with 65.7% accuracy, while epithelial Cx43 expression had no predictive capacity.
